# A Wolf in Hiding: Epilepsy and Post-ictal Psychosis As Unrecognized Presenting Features of Systemic Lupus Erythematosus

**DOI:** 10.7759/cureus.29577

**Published:** 2022-09-25

**Authors:** Serdar Akkol, Itamar Shapira, Norman Winn Gayle Seay, James T Houston

**Affiliations:** 1 Department of Neurology, University of Alabama at Birmingham School of Medicine, Birmingham, USA; 2 Department of Nephrology, University of Alabama at Birmingham, Birmingham, USA; 3 Department of Medicine, University of Alabama at Birmingham, Birmingham, USA; 4 Department of Neurology, University of Alabama at Birmingham, Birmingham, USA

**Keywords:** chronic kidney disease (ckd), glomerulonephritis, post-ictal psychosis, drug resistant epilepsy, neuropsychiatric sle

## Abstract

Systemic lupus erythematosus (SLE) is a chronic autoimmune disorder that affects multiple organ systems. Many patients present with neurological and psychiatric signs and symptoms at some point in the course of the disease. Here, we present a patient with neuropsychiatric SLE (NPSLE) who presented with long-standing and difficult-to-control epileptic seizures and post-ictal psychotic symptoms prior to the diagnosis of SLE. A 39-year-old patient with a ten-year history of uncontrolled epileptic seizures despite multiple medications and recent diagnosis of chronic kidney disease presented to the emergency department following multiple witnessed seizures. Her seizures were controlled following initial interventions and the patient was admitted to the hospital to control metabolic acidosis and hyperkalemia. Later, the patient developed psychosis with auditory hallucinations, combative behavior, and agitation which were controlled with restraints and sedatives. Initial serological and urinary studies revealed disturbances of multiple systems and triggered broad workup resulting in positive serological SLE markers. The patient was then started on immunosuppressive medications with prompt control of post-ictal psychosis. The patient was discharged with immunosuppressive regimen and control of her seizures. This case highlights that signs and symptoms of NPSLE may appear before the onset of SLE diagnosis. Additionally, our patient had long-standing epilepsy with post-ictal psychosis, which has not been reported in the literature before. We believe this case highlights the challenges in the diagnosis of NPSLE, the rapid control of seizures and/or psychosis with SLE treatment, and the necessity to broaden the differential diagnosis in atypical presentation of seizures and/or psychosis.

## Introduction

Systemic lupus erythematosus (SLE) is a chronic autoimmune disorder with a pathogenesis that is still unclear. SLE affects multiple organ systems and is hard to identify due to a multitude of nonspecific symptoms and signs [1]. SLE can affect joints, skin, kidney, heart, lungs, and the central nervous system (CNS), and most symptoms constellating the SLE diagnosis have been described more than 100 years ago [2].

CNS-related manifestations of SLE are commonly grouped under neuropsychiatric SLE (NPSLE) and range from headaches and cognitive impairment to psychosis, stroke, and epilepsy [3]. Epileptic seizures commonly occur in 4.6-12.5% of patients as part of SLE [4,5]. On the other hand, psychosis is a rare manifestation of the NPSLE [4,6,7]. Here, we present a case of a 39-year-old female with a ten-year history of uncontrolled epilepsy with post-ictal psychotic episodes and recently diagnosed chronic kidney disease. The patient was eventually diagnosed with SLE and responded well to immunotherapy.

## Case presentation

Our patient was a 39-year-old female with a past medical history significant for epilepsy, stage 3b chronic kidney disease (CKD), major depressive disorder, and generalized anxiety disorder, who presented after twelve reported seizures in 24 hours prior to admission, including a witnessed generalized tonic-clonic seizure in the ambulance triage area. She was subsequently given 2 mg of IV midazolam before being loaded with 1 g of IV levetiracetam. This presentation was similar to prior hospitalizations, as recently as three weeks prior, in which the patient was prescribed oxcarbazepine 300 mg twice daily at discharge. Given our patient’s presentation with a non-anion gap metabolic acidosis, hyperkalemia, and with concurrently increasing creatinine over the previous four months, she was admitted to the inpatient nephrology wards with neurology consultation.

She reported that her seizures initially began approximately ten years prior to her current presentation with no family history of SLE or epilepsy. The patient and caregiver described two seizure types. The first type was described as convulsive, sometimes only involving a single leg, accompanied by growling, as well as bowel and bladder incontinence and tongue biting. It would occur on average every four days, primarily at night, and would last five to ten minutes. These episodes were preceded by significant mood swings and were characterized by post-ictal generalized soreness, confusion, memory disturbance and language problems. The second seizure type involves behavioral arrest, manual automatisms, and loss of consciousness with falls. These happened at bi-weekly frequencies over the last five years. They were preceded by olfactory and visual auras and would result in post-ictal combative and agitated behavior. Prior anti-seizure medications include lacosamide, phenytoin, oxcarbazepine, and lorazepam; however, the patient reports repeatedly discontinuing these medications due to side effects.

**Figure 1:**
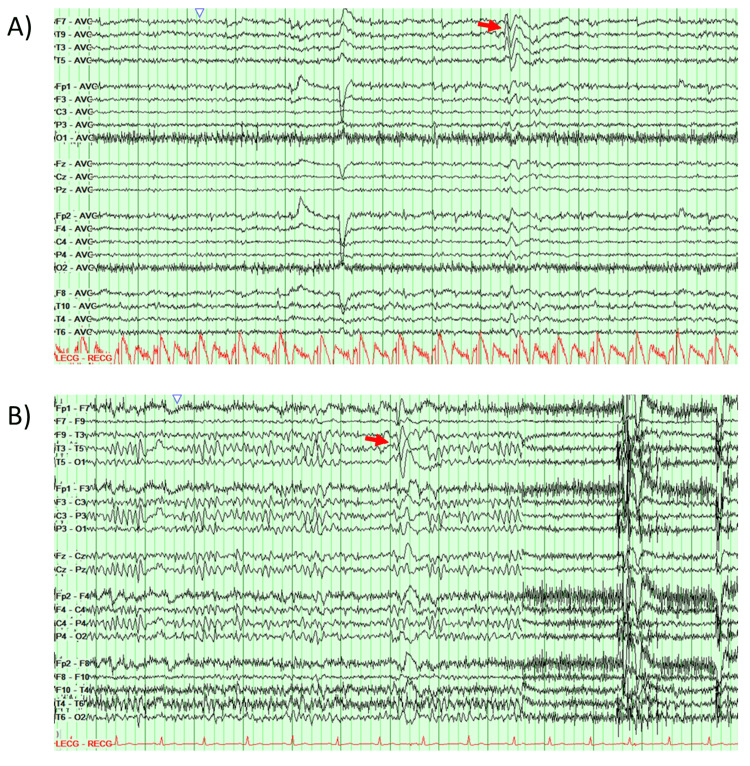
Example interictal discharges. During the long-term EEG recording of prior admissions, left fronto-temporal epileptiform discharges were captured. (A) shows bipolar montage of standard international 10-20 scalp EEG with sharp wave that is maximal on T9 and T3. (B) shows average montage recording with a different sharp wave that was maximal on F9-T3 and T3-T5 leads. Red arrows point out to the sharp waves at the maximal channel. Thick green vertical lines represent one-second intervals.

Prior routine electroencephalograms (EEGs) demonstrated sharp/slow waves over the left anterior temporal region and occasional sharp waves in right central and posterior regions. Previous admission to the epilepsy monitoring unit revealed intermittent, subtle, low voltage polymorphic slowing over the L temporal (lateral) chains as well as rare moderate to high voltage, spike/sharp and slow-wave discharges with maximal surface negativity over the L midtemporal region and a field extending into the L anterior temporal/frontotemporal region (Figure 1). No independent discharges were recorded over the right hemisphere.

Workup following admission was significant for worsening of CKD with serum creatinine of 2.5 mg/dL up from baseline of 2.1. Urinalysis showed 3+ proteinuria, and the urine albumin to creatinine ratio had increased significantly over the prior four months, from 600 mg/g to 2000 mg/g. Hematologic abnormalities were also noted, with bicytopenia including platelet counts about 100,000 per mm3 and hemoglobin levels around 7 g/dL, as well as direct antibody tests positive for warm agglutinin antibodies without signs of hemolysis. Given patient’s multiple findings, a workup for multisystem disease was initiated.

About two days after the patient’s presentation, nurses reported acute mental status change and agitation in the early hours of the morning, with the patient attempting to remove lines from her arms and elope. This was surprising, because she was calm and agreeable to further care, despite being somewhat confused during the prior day’s rounds. Now, the patient was hard to redirect by the physician, and later the patient attempted to elope with the patient unable to demonstrate capacity before attempting to bite providers. She was oriented only to self and location. The patient then required four-point restraints and sedation with diazepam 2 mg IV. Haloperidol 5mg IM was added after the patient chewed through two restraints. The patient’s husband was reached after this episode and stated that this behavior was consistent with post-ictal states that he had witnessed, voicing frustration that no solution had been found for these problems over the years. Psychiatrist saw the patient several hours after this event. She remained belligerent, attempting to spit at the physicians and seemingly responding to internal stimuli, consistent with post-ictal psychosis given the patient’s reported history of epilepsy. The patient remained altered on post-admission day three, refusing medications and requiring intramuscular hydralazine to maintain control of blood pressure. On that day, serological workup came back markedly positive for double-stranded DNA antibody (titer of >1:640) and antinuclear antibody (titer of >1:1280) prompting consultation with rheumatology and further workup of presumed SLE diagnosis. Her behavior and mentation improved somewhat, and the patient again participated in her care, later telling psychiatry consultants that she also experienced auditory hallucinations post-ictally.

Rheumatology recommended hydroxychloroquine (HCQ) 200 mg per oral, daily, and IV methylprednisolone 1 mg/kg/day while awaiting results of MRI of brain, lumbar puncture with cerebrospinal fluid (CSF) studies for standard CSF analysis, autoimmune encephalitis, anti-ribosomal P antibody, and oligoclonal bands and IgG index. After the first day of steroids and HCQ, the patient stated subjective improvement in her mood, behavior, and memory, better than she could ever recall. IV pulse steroid continued for a total of three days. MRI of the brain with contrast did not reveal any abnormal enhancement of the parenchyma, but CSF analysis was positive oligoclonal bands with normal cell counts, glucose, protein, and Mayo Clinic CSF panel. Further serological findings supported the diagnosis of SLE including positive antinuclear ribonucleoprotein antibodies and anti-Smith antibodies, low C3 level with normal C4, and negative anti-SSa, anti-SSb, and anti-NMDA receptor antibody. A renal biopsy was obtained revealing focal sclerosing and membranous glomerulonephritis (International Society of Nephrology and the Renal Pathology Society (ISN/RPS) class III and class V).

Per patient wishes, she was given a medication regimen including HCQ 400 mg per oral (PO) twice-daily (BID), mycophenolate mofetil 1500 mg BID, prednisone 40 mg PO daily (course of 30 days), oxcarbazepine 300 mg PO BID, hydralazine 50 mg PO BID, amlodipine 10 mg PO daily, labetalol 200 mg TID (three times a day), and losartan 100 mg PO daily. Patient was seen by his neurologist two weeks after her discharge, it was found that she did not have any episodes of seizures or psychosis.

## Discussion

SLE is a chronic autoimmune disorder with a multitude of clinical presentations and is hard to recognize. Symptoms are vague and gradual in their onset and appear at different times in the course of the disease and sometimes are not recognized as part of the disease [1,8]. This, unfortunately, delays the diagnosis and treatment of SLE. Thus, it is important to investigate signs and symptoms that could constellate SLE and have responded inadequately to treatment.

NPSLE manifestations appear in 10-80% of the patients with SLE [9]. Although NPSLE has been recognized by the American College of Rheumatology and the European League Against Rheumatism (ACR/EULAR) 2019 edition of SLE classification [1], some of the manifestations of the disease may appear years before diagnosis [10]. These manifestations can occur in the absence of serologic or other systemic markers of SLE, thus resulting in progression of SLE and unsuccessful therapeutic interventions, as was the case in our patient with many unsuccessful trials of anti-epileptic drugs resulting in nonadherence with medications and management [11,12].

Epileptic seizures commonly develop early after the diagnosis of SLE [5,11,12]. In one study, seizures presented with an interval of 0.14 (−0.50 to 7.57) years from the time of SLE diagnosis to the first seizure. In rare circumstances, status epilepticus may be the initial presenting symptom [13,14] and epilepsy may appear years before the diagnosis, as was the case in our patient [12,15]. It remains unclear if NPSLE and epileptic seizures in our patient were related; we believe seizures were part of NPSLE since the post-ictal psychosis was rapidly aborted by steroids and HCQ. Additionally, during an early clinic follow-up, she mentioned that she did not have seizures since discharge from hospital, further supporting the relation between seizures and SLE. Although epilepsy is a relatively common feature of NPSLE, the underlying mechanisms that lead to seizures are yet to be understood. It has been hypothesized that ischemic vascular disease or auto-antibodies (e.g. antiphospholipid antibodies) are the possible cause(s) for seizures [15], however this remains unclear [16]. EEG recordings of NPSLE cases have shown that interictal abnormalities are predominant in temporal cortex and asymmetric [15].

Psychiatric symptoms like delirium, anxiety, or mood disorders may develop at any stage of the disease [7]. Previous studies have shown that psychosis was more prevalent with patients with epileptic seizures than those without seizures [4]. In our case, psychotic episodes followed the seizures. We believe this is the first SLE case with epileptic seizures and post-ictal psychosis.

Management of NPSLE is challenging as the evidence for different treatment choices is unclear [3,7]. Initial treatment involves both symptomatic treatment and the control of the autoimmune disease process with disease-modifying agents such as corticosteroids and anti-malarial agents, for example, anti-epileptic drugs for seizures and anxiolytics, antidepressants, mood-stabilizers, or antipsychotics for psychiatric manifestations. Although it might seem counterintuitive, many neurological manifestations of NPSLE can be controlled with corticosteroids, antimalarial, and other immunosuppressive agents [3]. In a prospective cohort, anti-epileptic drugs given for SLE-related seizures were discontinued more frequently than for non-SLE-related seizures [5]. A previous case study showed the resolution of status epilepticus in response to IV methylprednisolone in an NPSLE case [14]. Similarly, our patient was also started on methylprednisolone and HCQ with prompt control of the post-ictal psychosis.

## Conclusions

In conclusion, this case presentation emphasizes the importance of diagnosis of NPSLE and the rapid control of seizures and psychosis with SLE treatment. We believe that atypical presentation of seizures and/or psychosis should prompt physicians to consider alternative explanations, like SLE, a disease whose specific symptoms may appear later in the disease course and whose symptoms and disease progression can be controlled with immunosuppressive drugs.
